# Psychosocial aspects and support networks associated with disability in two longevous populations in Brazil: a cross-sectional study

**DOI:** 10.1186/s12877-022-02810-4

**Published:** 2022-02-09

**Authors:** Júlia Cristina Leite Nóbrega, Juliana Barbosa Medeiros, Javanna Lacerda Gomes da Silva Freitas, Jaíza M. M. Silva, Raisa Fernandes Mariz Simões, Ricardo Olinda, Jair Lício de Ferreira Santos, Tarciana Nobre Menezes, Yeda Aparecida de Oliveira Duarte, Mayana Zatz, David Matheson, Silvana Santos

**Affiliations:** 1 Public Health Program, Universidade Estadual da Paraíba (UEPB), Campina Grande, Brazil; 2 Department of Statistics, Universidade Estadual da Paraíba (UEPB), Campina Grande, Brazil; 3 Department of Social Medicine, Universidade de São Paulo (USP), São Paulo, Brazil; 4 Department of Physical Therapy, Universidade Estadual da Paraíba (UEPB), Campina Grande, Brazil; 5 Department of Medical Surgical Nursing, School of Nursing, Universidade de São Paulo, São Paulo, Brazil; 6 Human Genome Studies Center, Universidade de São Paulo (USP), São Paulo, Brazil; 7 Faculty of Education, Health and Wellbeing, University of Wolverhampton, Wolverhampton, UK; 8 Department of Biology, Universidade Estadual da Paraíba (UEPB), Campina Grande, Brazil

**Keywords:** Activities of daily living, Physical functional performance, Psychology, Social support, Ageing, Aged, 80 and over

## Abstract

**Background:**

Among the oldest old, aged 80 years and over, the prevalence of disability is higher than in other age groups and can be considered a predictor of mortality.

**Objective:**

To evaluate how psychosocial aspects and support networks influence the disability of these oldest-old individuals, performing a comparison between two longevous populations, one living in one of the poorest regions of Brazil, in the backlands of Paraíba, and another living in one of the largest urban centres in Latin America.

**Method:**

A cross-sectional study in which 417 oldest-old persons aged 80 years and older were interviewed, with data collected through the “Health, Welfare and Ageing” survey conducted in two Brazilian cities. Disability was assessed by reporting the need for assistance in Activities of Daily Living (ADLs) and Instrumental Activities of Daily Living (IADLs). Bivariate and multiple analyses were performed using R statistical software.

**Results:**

Food insufficiency in the first years of life had negative repercussions on the disability of oldest old people living in the northeast. On the other hand, in this region, older people have a higher rate of support and live longer with their peers, which may contribute to reducing feelings of loneliness, depressive symptoms, and worse self-perception of health. In the Southeast, financial constraints, subjective poverty, and unmet needs may favour the development of functional limitations between long-lived people.

**Conclusion:**

Our findings indicate that regional differences in Brazil may influence the disability of older people aged 80 and older. In northeast Brazil, having no partner may contribute to disability for ADLs and IADLs; while, in the longevous population of São Paulo, having a worse self-rated health may contribute to disability for IADLs.

**Supplementary Information:**

The online version contains supplementary material available at 10.1186/s12877-022-02810-4.

## Background

Functional performance in people aged 80 years and older is a strong predictor of mortality, hospitalisation, and disability [1]. During the ageing process, there is an increasing risk of disability, especially at older ages. Disability has been defined as acquired difficulty in performing basic everyday tasks or more complex tasks needed for independent living, results from the interaction of multidimensional factors [2]. Disability can be assessed by the need for partial, or total, help for at least one of the daily activities investigated including Activities of Daily Living (ADLs) and Instrumental Activities of Daily Living (IADLs) [3]. ADLs are linked to the individual's self-care such as eating, bathing, and dressing. The IADLs encompass tasks more related to the social participation of the subject, such as shopping, answering the telephone and using means of transportation [4].

A senior faces the social and health challenges of ageing, including the greater likelihood of difficulties in performing activities of daily living and the existential challenges that arise as they approach the end of life. In Brazil, as in other Latin American countries, these concerns are experienced in a society that is not yet fully prepared for a large population of seniors. Although the Brazilian public health system offers universal access to free treatment, public health still faces some challenges including the scarcity of resources to provide health services for seniors that are appropriate in quantity and quality [5]. At the same time, disabilities resulting in functional limitations restrict the seniors and their families, leading to a greater demand for care and health services to assist with ADLs and IADLs [6].

Brazil is a continental country with regional asymmetries and inequalities, as well as sociocultural and psychosocial differences which may influence disability. In rural and poor communities of north-eastern Brazil, such as Brejo dos Santos, there is a high prevalence of consanguineous marriages associated with the founder effect [7], which can increase the social support among family members. Also, these communities have greater homogeneity in income and lifestyle when compared to populations living in large urban centres, such as São Paulo. The literature has shown that psychosocial aspects including depression [2, 8–10], self-rated health [8, 10], subjective well-being [11], life satisfaction [12], loneliness [8, 9, 13] and isolation [14, 15] seem to have a very important effect on disability, especially among the oldest old. Another aspect that influences longevity is human relationships and cohabitation arrangements as they seem to have an impact on health [16], so the social support network should be studied.

In this paper, the objective was to make a comparison between two longevous populations, one living in one of the poorest regions of Brazil, in the hinterlands of Paraíba, and another living in one of the largest urban centres in Latin America, with the purpose of evaluating how psychosocial aspects and support networks influence the disability of these longevous populations.

## Method

This is a cross-sectional, analytical study with a quantitative approach in which data from the oldest-old aged 80 years and older living in Brejo dos Santos/PB and São Paulo/SP were evaluated. Data were collected through the questionnaire of the Health, Well-being and Ageing Study (*Saúde, Bem-Estar e Envelhecimento*—SABE) [17, 18] administered by trained interviewers and conducted in the homes of the oldest-old. In Brejo dos Santos-PB, data collection occurred in May 2017 and in São Paulo-SP between March and June 2016.

Potential participants were informed about the objectives of the study and their agreement was obtained. No data was collected until after they signed the Informed Consent Form, following the ethical aspects of research involving human subjects, as ruled under Resolution No. 466/2012 of the National Health Council of the Ministry of Health of Brazil. The SABE-PB study was approved by the Research Ethics Committee of the State University of Paraíba. The SABE-SP study was approved by the Research Ethics Committee of the Public Health College at the University of São Paulo.

The population of SABE-PB consisted of 179 oldest-old aged 80 years and over, residing in the town of Brejo dos Santos/PB, Brazil, belonging to the total of 188 listed by the Municipal Health Secretariat. Inclusion criteria were men and women, 80 years old and over and residents of the town. Losses occurred from refusals, migration to another city, and other reasons (4.9 per cent). In this study, all oldest-old persons living in the city were invited to be included in the study.

Oldest-old of SABE-SP were selected through a representative probabilistic sample of the urban population of São Paulo/SP aged 60 years or older. A detailed description of the SABE-SP study design and sampling process was previously published [17, 18]. For this cross-sectional analysis, the sample was restricted to 238 oldest-old aged 80 years or older who participated in the fourth SABE-SP.

The outcome variable was disability and was assessed using two outcomes that were analysed by reporting need for help in ADLs and IADLs according to the Katz et al. [19] and Lawton et al. [20] indexes. Questions about ADLs included walking, dressing, bathing, personal hygiene, combing hair, eating alone, lying down and getting up from bed and chairs, and going to the bathroom. The IADLs were: preparing a hot meal, taking care of their own money, using transportation, shopping, phoning, doing light housework, and taking medicine. Participants were asked if they received help for each activity and could choose to answer: “yes” or “no”. Those oldest-old who answered “no” were classified as having no disability while those who answered “yes” for at least one of the activities were classified as having disability.

Exposure variables included sociodemographic factors, health conditions, and psychosocial aspects. The following variables were selected: gender (female and male), age (categorised as 80 to 89 years, and 90 years and older), marital status (“with partner” for those who were married and cohabitating, and “no partner” for widowed, divorced, separated and single), literacy (“yes” for those who went to school and could write and read, and “no” for those who did not meet both these two criteria), income (≤ 1 minimum wage and > 1 minimum wage), sufficient income (whether the oldest old consider their income sufficient for their expenses or not), insufficient food up to the age of 15 (“yes” or “no”), self-rated health (categorised as good, regular and bad), subjective well-being (better and worse) [21], overall life satisfaction (highest and lowest) [22], depressive symptoms (yes/no) [23], loneliness (yes/no), isolation (yes/no), receiving social support (yes/no), offering social support (yes/no), co-residence (yes/no), conviviality most of the time (children and/or adolescents, adults and seniors) and number of members of the network (only one, between two and four, and five or more).

Some of these variables are subjective and therefore cannot be answered by substitute informants, so it was decided to check suggestive cognitive decline through the Mini-Mental State Examination (MMSE), which was originally composed of questions grouped into seven categories to assess specific cognitive functions: time orientation, place orientation, three-word registration, attention and calculation, three-word recall, language, and visual constructive ability [24]. Oldest-old with suggestive cognitive decline were excluded from the analytical analysis.

To assess cognitive function, the MMSE was modified and validated for the SABE Study, due to the low educational level of the elderly population in South America. The instrument has three items that are less dependent on education level and the cut-off point is 12 points or less. Score below 12 was considered suggestive of cognitive deficit [25].

For processing and analysis, and in order to obtain better consistency the data were tabulated in the Epidata 3.1 double-entry program. Afterwards, they were analysed using R statistical software (R CORE TEAM, 2018), consisting of bivariate and multiple logistic regression analysis of the data. In the bivariate analysis the Pearson's chi-square test, the Fisher's exact test in cases where one of the frequencies was less than 5 were used, and the selected measure of association was the odds ratio (OR). For multiple analysis, the initial logistic regression model was obtained with all variables taking as measures of association the OR and 95% confidence intervals (95% CI). The adjustment variables that presented p ≤ 0.20 in the initial model were included in the final multiple analyses and in the interpretation of the results, *p* < 0.05 was considered as a statistically significant association.

## Results

The populations of Brejo dos Santos and São Paulo have different socio-demographic profiles, as shown in Table 1. In Brejo dos Santos, 54.70 percent of the 179 oldest-old are women, with an average of 85.47 years (± 5, *n* = 33) with a range of 80 to 102 years. Regarding marital status, 49.70 per cent of the long-lived were widowed and the average income is low, with 67.00 percent receiving up to one minimum wage while 62.00 per cent of them were not literate. In São Paulo, there are more women, reaching 70.60 percent of the 238 oldest-old sample. Age ranged from 80 to 101 years, with a mean of 86.81 years (± 4.73) and 69.70 per cent of the long-lived were widowed. Most (58.10 per cent) received more than one minimum wage and only 21.30 per cent were not literate (Table 1). The Supplementary Table 1 shows the number of ADLs and IADLs with support.

**Table 1:** Distribution of the oldest-old according to socioeconomic and demographic characteristics. psychosocial aspects and support network characteristics. SABE Study. Brejo dos Santos/PB. Brazil. 2017 (*n* = 179) and São Paulo/SP. Brazil. 2015 (*n* = 238)

Variables	SABE-PB		SABE-SP	
	**n**	**%**	**n**	**%**
**Gender**				
Women	98	54.70	168	70.60
Men	81	45.30	70	29.40
**Age**				
80 to 89 years old	141	78.80	173	72.70
90 years old and more	38	21.20	65	27.30
**Marital status**				
Married	76	42.50	48	20.20
Amused	1	0.60	2	0.80
Divorced/Separated	7	3.90	12	5.10
Widowed	89	49.70	166	69.70
Single	6	3.40	10	4.20
**Literacy**				
Yes	68	38.00	181	78.70
No	111	62.00	49	21.30
**Food insufficiency in the first 15 years of life**				
Yes	67	41.40	33	14.80
No	95	58.60	190	85.20
**Income**				
≤ 1 minimum wage	118	67.00	88	41.90
> 1 minimum wage	58	33.00	122	58.10
**Income Sufficiency**				
Yes	108	61.00	135	60.00
No	69	39.00	90	40.00
**Self-reported health**				
Good/very good	51	38.90	93	43.50
Regular	66	50.40	93	43.50
Bad/very bad	14	10.70	28	13.10
**Subjective well-being**				
Worse	31	23.10	43	21.30
Better	103	76.90	159	78.70
**Global satisfaction with life**				
Lower	18	12.90	43	19.20
Higher	122	87.10	181	80.80
**Depressive symptoms**				
Yes	38	42.20	118	49.60
No	52	57.80	120	50.40
**Diagnostic of depression**				
Yes	12	6.80	39	16.80
No	165	93.20	193	83.20
**Medicines for depression**				
Yes	7	3.95	29	78.40
No	170	96.05	8	21.60
**Loneliness**				
Yes	78	54.90	84	41.20
No	64	45.10	120	58.80
**Social isolation**				
Yes	50	35.70	49	24.00
No	90	64.30	155	76.00
**Receiving social support**				
No	34	19.00	73	30.80
Yes	145	81.00	164	69.20
**Offering social support**				
No	41	22.90	95	40.10
Yes	138	77.10	142	59.90
**Co residence**				
No	25	14.00	62	26.60
Yes	154	86.00	171	73.40
**Home arrangement**				
Living alone	25	14.00	62	26.60
Spouse only	37	20.70	29	12.40
Offspring	45	25.10	64	27.50
Offspring and grandchildren	32	17.90	41	17.60
Other arrangements	40	22.30	37	15.90
**Conviviality most of the time**				
Living alone	3	1.90	75	32.50
Children and/or adolescents	7	4.50	12	5.20
Seniors	83	53.20	77	33.30
Adults	63	40.40	67	29.00
**Nº of network members**				
Living alone	25	14.00	62	26.60
Between 2 and 4	130	72.60	144	61.80
5 or more	24	13.40	27	11.60
**Disability for ADLs**				
Yes	56	31.50	65	27.40
No	122	68.50	172	72.60
**Disability for AIVDs**				
Yes	156	87.20	166	70.00
No	23	12.80	71	30.00

*SABE* Health, well-being and ageing, *ADLs* Activities of daily living, *IADLs* Instrumental activities of daily living

Table 2 shows bivariate analysis for association between socioeconomic, demographic, psychosocial and support network characteristics with disability on ADLs. It was observed that, in the São Paulo sample, there was a significant difference for most of the analysed variables, which did not occur in the study population of Brejo dos Santos. For example, concerning economic aspects, most long-living people living in Brejo dos Santos receive a rural retirement pension of a minimum wage and they are relatively satisfied with this income; no association of these factors with disability was observed, as also occurred with the São Paulo sample. On the other hand, in Brejo dos Santos, an association between disability and insufficient food up to 15 years of age and marital status was observed, aspects not observed in São Paulo.

**Table 2:** Bivariate association between demographic socioeconomic characteristics, psychosocial aspects, and support networks to ADLs disability. SABE Study, Brejo dos Santos/PB, Brazil, 2017 and São Paulo/SP, Brazil, 2015

Variables	Disability for ADLs									
	**SABE-PB**					**SABE-SP**				
	**Yes**	**No**	**OR**	**CI**	***p***	**Yes**	**No**	**OR**	**CI**	***p***
	**n(%)**	**n(%)**	**crude**	**95%**		**n(%)**	**n(%)**	**crude**	**95%**	
**Gender**					**0.04**					**0.011**
Women	37(20.79)	61(34.27)	1.94	1.009–3.75		54(22.78)	114(48.1)	2.49	1.21–5.13	
Men	19(10.67)	61(34.27)	1.0	1.0		11(4.64)	58(24.47)	1.0	1.0	
**Age**					**0.006**					** < 0.001**
90 years old and more	19(10.67)	19(10.67)	2.78	1.33–5.82		31(13.08)	33(13.92)	3.84	2.07–7.11	
80–89 years old	37(20.79)	103(57.87)	1.0	1.0		34(14.35)	139(58.65)	1.0	1.0	
**Marital status**					**0.04**					0.11
No partner	38(21.35)	63(35.39)	1.97	1.018–3.84		56(23.63)	132(55.7)	1.88	0.85–4.14	
With a partner	18(10.11)	59(33.15)	1.0	1.0		9(3.80)	40(16.88)	1.0	1.0	
**Literacy**					0.14					** < 0.001**
No	39(21.91)	71(39.89)	1.64	0.84–3.23		22(9.61)	27(11.79)	3.25	1.66–6.37	
Yes	17(9.55)	51(28.65)	1.0	1.0		36(15.72)	144(62.88)	1.0	1.0	
**Income**					0.1					** < 0.001**
≤ 1 minimum wage	33(18.86)	85(48.57)	0.57	0.29–1.11		35(16.67)	53(25.24)	3.36	1.77–6.39	
> 1 minimum wage	23(13.14)	34(19.43)	1.0	1.0		20(9.52)	102(48.57)	1.0	1.0	
**Income sufficiency**					0.41					**0.007**
No	24(13.64)	45(25.57)	1.3	0.68–2.50		31(13.78)	59(26.22)	2.31	1.25–4.27	
Yes	31(17.61)	76(43.18)	1.0	1.0		25(11.11)	110(48.89)	1.0	1.0	
**Food insufficiency up to 15 years old**					**0.02**					0.51
Yes	24(14.91)	43(26.71)	2.2	1.08–4.47		9(4.04)	24(10.76)	1.32	0.57–3.05	
No	19(11.80)	75(46.58)	1.0	1.0		42(18.83)	148(66.37)	1.0	1.0	
**Self-reported health**					0.34					**0.012**
Bad	2(1.54)	12(9.23)	1.5	0.26–8.71		12(5.61)	16(7.48)	3.61	1.44–9.08	
Regular	13(10)	53(40.77)	2.21	0.73–6.67		15(7.01)	78(36.45)	0.93	0.43–2	
Good	5(3.85)	45(34.62)	1.0	1.0		16(7.48)	77(35.98)	1.0	1.0	
**Subjective well-being**					0.19					** < 0.001**
Worse	8(6.02)	23(17.29)	1.87	0.71–4.90		17(8.42)	26(12.87)	5.12	2.33–11.21	
Better	16(12.03)	86(64.66)	1.0	1.0		18(8.91)	141(69.8)	1.0	1.0	
**Global satisfaction with life**					0.68					**0.009**
Lower	4(2.88)	14(10.07)	1.28	0.38–4.28		17(7.59)	26(11.61)	2.54	1.25–5.17	
Higher	22(15.83)	99(71.22)	1.0	1.0		37(16.52)	144(64.29)	1.0	1.0	
**Depressive symptoms**					0.62					**0.03**
Yes	5(5.62)	33(37.08)	1.39	0.37–5.20		12(7.1)	37(21.89)	2.45	1.04–5.78	
No	5(5.62)	46(51.69)	1.0	1.0		14(8.28)	106(62.72)	1.0	1.0	
**Loneliness**					0.94					**0.035**
Yes	14(9.93)	64(45.39)	1.03	0.43–2.46		20(9.80)	64(31.37)	2.18	1.04–4.57	
No	11(7.80)	52(36.88)	1.0	1.0		15(7.35)	105(51.47)	1.0	1.0	
**Social isolation**					0.64					**0.004**
Yes	10(7.19)	40(28.78)	1.23	0.50–2.99		15(7.35)	34(16.67)	2.97	1.38–6.41	
No	15(10.79)	74(53.24)	1.0	1.0		20(9.80)	135(66.18)	1.0	1.0	
**Receiving social support**					**0.02**					**0.007**
No	5(2.81)	28(15.73)	0.32	0.12–0.90		11(4.66)	61(25.85)	0.37	0.18–0.77	
Yes	51(28.65)	94(52.81)	1.0	1.0		53(22.46)	111(47.03)	1.0	1.0	
**Offering social support**					0.58					0.17
No	14(7.87)	26(14.61)	1.23	0.58–2.59		30(12.71)	64(27.12)	1.48	0.83–2.66	
Yes	42(23.6)	96(53.93)	1.0	1.0		34(14.41)	108(45.76)	1.0	1.0	
**Co residence**					**0.008**^**a**^					**0.002**
No	2(1.12)	22(12.36)	0.16	0.38–0.74		8(3.43)	54(23.18)	0.29	0.13–0.66	
Yes	54(30.34)	100(56.18)	1.0	1.0		57(24.46)	114(48.93)	1.0	1.0	
**Home arrangement**					** < 0.001**					**0.015**
Living alone	2(1.12)	22(12.36)	0.19	0.04–0.93		8(3.43)	54(23.18)	0.27	0.10–0.75	
Spouse only	8(4.49)	29(16.29)	0.57	0.21–1.6		6(2.58)	23(9.87)	0.48	0.16–1.48	
Offspring	13(7.30)	32(17.98)	0.84	0.33–2.13		24(10.30)	40(17.17)	1.11	0.48–2.58	
Offspring and grandchildren	20(11.24)	12(6.74)	3.46	1.31–9.171		14(6.01)	27(11.59)	0.96	0.38–2.44	
Other arrangements	13(7.30)	27(15.17)	1.0	1.0		13(5.58)	24(10.3)	1.0	1.0	
**Conviviality most of the time**					0.33					** < 0.001**
Living alone	0(0)	3(1.92)	-	-		6(2.60)	69(29.87)	0.11	0.04–0.3	
Children and/or adolescents	2(1.28)	5(3.21)	0.61	0.11–3.38		4(1.73)	8(3.46)	0.66	0.18–2.39	
Seniors	28(17.95)	55(35.26)	0.77	0.39–1.53		26(11.26)	51(22.08)	0.67	0.34–1.31	
Adults	25(16.03)	38(24.36)	1.0	1.0		29(12.55)	38(16.45)	1.0	1.0	
**Nº of network members**					**0.012**					**0.005**
Living alone	2(1.12)	22(12.36)	0.13	0.02–0.67		8(3.43)	54(23.18)	0.25	0.09–0.74	
Between 2 and 4	44(24.72)	86(48.31)	0.72	0.29–1.74		47(20.17)	97(41.63)	0.82	0.35–1.94	
5 or more	10(5.62)	14(7.87)	1.0	1.0		10(4.29)	17(7.3)	1.0	1.0	

*SABE* Health, Well-being and Ageing, *ADLs* Activities of daily living

^a^Fisher exact test

Most psychosocial variables showed no association with dependence for ADLs in the sample of Brejo dos Santos when compared to São Paulo, such as subjective well-being, depressive symptoms or feelings of loneliness. On the other hand, some variables such as family arrangement, co-residence and having social support show association in both investigated populations, highlighting the relationship between the oldest-old support network and its ability to perform activities of daily living (Table 2).

The bivariate analysis of the associated factors concerning disability in IADLs is presented in Table 3. There was no association with gender, self-rated health, life satisfaction, loneliness and social support network for both populations studied. Only in Brejo dos Santos, the disability for IADLs showed association with marital status, and with insufficient food, and the oldest-old who were starved in childhood were more dependent. For all other psychosocial variables, there was an association only in the population of São Paulo, showing very different profiles concerning the factors that influence the performance of IADLs. The Supplementary Table 1 shows a comparison between the two populations in relation to the number of ADLs and IADLs with support.

**Table 3:** Bivariate association between demographic socioeconomic characteristics, psychosocial aspects, and support networks to IADLs disability. SABE Study, Brejo dos Santos/PB, Brazil, 2017 and São Paulo/SP, Brazil, 2015

Variables	Disability on IADLs									
	**SABE-PB**					**SABE-SP**				
	**Yes**	**No**	**OR**	**IC**	***p***	**Yes**	**No**	**OR**	**IC**	***p***
	**n(%)**	**n(%)**	**bruto**	**95%**		**n(%)**	**n(%)**	**bruto**	**95%**	
**Gender**					0.52					0.46
Women	84(46.93)	14(7.82)	0.75	0.30–1.83		120(50.63)	48(20.25)	1.25	0.68–2.28	
Men	72(40.22)	9(5.03)	1.0	1.0		46(19.41)	23(9.7)	1.0	1.0	
**Age**					**0.005**^**a**^					** < 0.001**
90 years and more	38(21.23)	0(0.0)	1.19	1.11–1.28		58(24.47)	6(2.53)	5.81	2.37–14.23	
80–89 year old	118(65.92)	23(12.85)	1.0	1.0		108(45.57)	65(27.43)	1.0	1.0	
**Marital Status**					**0.006**					0.81
No partner	95(53.07)	7(3.91)	3.56	1.38–9.15		131(55.27)	57(24.05)	0.91	0.46–1.83	
With a partner	61(34.08)	16(8.94)	1.0	1.0		35(14.77)	14(5.91)	1.0	1.0	
**Literacy**					0.13					0.07
No	100(55.87)	11(6.15)	1.94	0.80–4.70		39(17.03)	10(4.37)	1.99	0.93–4.27	
Yes	56(31.28)	12(6.7)	1.0	1.0		119(51.97)	61(26.64)	1.0	1.0	
**Income**					**0.03**					** < 0.001**
≤ 1 minimum wage	99(56.25)	19(10.8)	0.28	0.08–1.0		72(34.29)	16(7.62)	3.23	1.68–6.19	
> 1 minimum age	55(31.25)	3(1.7)	1.0	1.0		71(33.81)	51(24.29)	1.0	1.0	
**Income Sufficiency**					0.63					**0.001**
No	59(33.33)	10(5.65)	0.8	0.33–1.95		73(32.44)	17(7.56)	2.86	1.52–5.37	
Yes	95(53.67)	13(7.34)	1.0	1.0		81(36)	54(24)	1.0	1.0	
**Food insufficiency up to 15 years old**					**0.012**					0.54
Yes	63(38.89)	4(2.47)	3.93	1.27–12.17		24(10.76)	9(4.04)	1.29	0.56–2.94	
No	76(46.91)	19(11.73)	1.0	1.0		128(57.40)	62(27.8)	1.0	1.0	
**Self-reported health**					0.36					0.13
Bad	12(9.16)	2(1.53)	1.85	0.36–9.43		22(10.28)	37(17.29)	2.42	0.9–6.54	
Regular	57(43.51)	9(6.87)	1.95	0.75–5.07		65(30.37)	28(13.08)	1.53	0.84–2.82	
Good	39(29.77)	12(9.16)	1.0	1.0		56(26.17)	37(17.29)	1.0	1.0	
**Subjective well-being**					0.1					**0.01**
Worse	29(21.64)	2(1.49)	3.28	0.72–14.95		35(17.33)	8(3.96)	2.79	1.21–6.42	
Better	84(62.69)	19(14.18)	1.0	1.0		97(48.02)	62(30.69)	1.0	1.0	
**Global satisfaction with life**					0.51					0.091
Lower	16(11.43)	2(1.43)	1.66	0.35–7.78		34(15.18)	9(4.02)	1.96	0.88–4.36	
Higher	101(72.14)	21(15)	1.0	1.0		119(53.12)	62(27.68)	1.0	1.0	
**Depressive symptoms**					0.39					**0.02**
Yes	32(35.56)	6(6.67)	1.6	0.54–4.73		38(22.49)	11(6.51)	2.46	1.15–5.29	
No	40(44.44)	12(13.33)	1.0	1.0		70(41.42)	50(29.59)	1.0	1.0	
**Loneliness**					0.37					0.2
Yes	64(45.07)	14(9.86)	0.65	0.25–1.67		59(28.92)	25(12.25)	1.46	0.80–2.66	
No	56(39.44)	8(5.63)	1.0	1.0		74(36.27)	46(22.55)	1.0	1.0	
**Social isolation**					0.36					**0.006**
Yes	44(31.43)	6(4.29)	1.58	0.57–4.35		40(19.61)	9(4.41)	2.96	1.34–6.53	
No	74(52.86)	16(11.43)	1.0	1.0		93(45.59)	62(30.39)	1.0	1.0	
**Receiving social support**					0.83					** < 0.001**
No	30(16.76)	4(2.23)	1.13	0.35–3.57		39(16.53)	33(13.98)	0.35	0.19–0.64	
Yes	126(70.39)	19(10.61)	1.0	1.0		126(53.39)	38(16.1)	1.0	1.0	
**Offering social support**					0.08					0.17
No	39(21.79)	2(1.12)	3.5	0.78–15.60		61(25.85)	33(13.98)	0.67	0.38–1.18	
Yes	117(65.36)	21(11.73)	1.0	1.0		104(44.07)	38(16.1)	1.0	1.0	
**Co residence**					0.43					** < 0.001**
No	23(12.85)	2(1.12)	1.81	0.39–8.27		31(13.30)	31(13.30)	0.29	0.16–0.54	
Yes	133(74.3)	21(11.73)	1.0	1.0		132(56.65)	39(16.74)	1.0	1.0	
**Home arrangement**					**0.002**					** < 0.001**
Living alone	23(12.85)	2(1.12)	0.93	0.14–6.01		31(13.30)	31(13.30)	0.19	0.07–0.53	
Spouse only	27(15.08)	10(5.59)	0.22	0.05–0.87		20(8.58)	9(3.86)	0.43	0.13–1.39	
Offspring	37(20.67)	8(4.47)	0.37	0.09–1.53		46(19.74)	18(7.73)	0.49	0.18–1.39	
Offspring and Children	32(17.88)	0(0.0)	-	-		35(15.02)	6(2.58)	1.13	0.33–3.86	
Other arrangements	37(20.67)	3(1.68)	1.0	1.0		31(13.30)	6(2.58)	1.0	1.0	
**Conviviality most of the time**					0.07					** < 0.001**
Living alone	2(1.28)	1(0.64)	0.17	0.01–2.25		36(15.58)	39(16.88)	0.22	0.1–0.47	
Children and/or adolescents	7(4.49)	0(0.0)	-	-		11(4.76)	1(0.43)	2.65	0.31–22.37	
Seniors	67(42.95)	16(10.26)	0.36	0.12–1.05		61(26.41)	16(6.93)	0.92	0.4–2.08	
Adults	58(37.18)	5(3.21)	1.0	1.0		54(23.38)	13(5.63)	1.0	1.0	
**Nº of network members**					0.51					** < 0.001**
Living alone	23(12.85)	2(1.12)	3.75	1.03–16.43		31(13.30)	31(13.30)	0.74	0.26–2.09	
Between 2 e 4	111(62.01)	19(10.61)	2.23	0.77–8.27		110(47.21)	34(14.59)	0.23	0.08–0.68	
5 or more	22(12.29)	2(1.12)	1.0	1.0		22(9.44)	5(2.15)	1.0	1.0	

*SABE* Health, Well-being and Ageing, *ADLs* Activities of daily living

^a^Fisher exact test

Table 4 presents the results of the multiple logistic regression analysis, highlighting the variables that influence the disability of the oldest old. For oldest-old that lived in São Paulo, having an income of less than or equal to one minimum wage worsens the disability 5.61 times for ADLs and 2.38 times for IADLs, showing how inequalities can influence the disability among seniors. In this urban population, those with a worse perception of health were 10.54 times more likely to have disability for ADLs and, being 90 years old or older increases in 4.59 the chances of developing disability for IADLs. In the rural community of Brejo dos Santos, gender was an independently associated factor with disability for IADLs indicating that being women was a protective factor and those who had no partner were 2.87 more likely to have a disability for ADLs and 9.31 more likely to have a disability for IADLs.

**Table 4:** Multiple logistic regression model for the association between socioeconomic and demographic characteristics, psychosocial aspects and support networks on disability of the oldest-old. SABE Study, Brejo dos Santos/PB, Brazil, 2017 and São Paulo/SP, Brazil, 2015

Variables	Disability							
	**ADLs**				**IADLs**			
	**SABE-PB**		**SABE-SP**		**SABE-PB**		**SABE-SP**	
	**ORadj (CI 95%)**	***p***	**ORadj (CI 95%)**	***p***	**ORadj (CI 95%)**	***p***	**ORadj (CI 95%)**	***p***
**Gender**				0.56		**0.01**		
Women	-		0.66 (0.15–2.79)		0.24 (0.07–0.78)		-	
Men	-		1.0		1.0		-	
**Age**								** < 0.001**
90 years old and more	-		-		-		4.59 (1.77–11.89)	
80–89 years old	-		-		-		1.0	
**Marital status**		**0.04**		0.4		** < 0.001**		
No partner	2.87 (0.96–8.59)		3.02 (0.21–43.77)		9.31 (2.09–41.54)		-	
With a partner	1.0		1.0		1.0		-	
**Income**				**0.01**				**0.01**
≤ 1 minimum wage	-		5.61 (1.39–22.64)		-		2.38 (1.14–4.97)	
> 1 minimum wage	-		1.0		-		1.0	
**Income sufficiency**								0.05
No	-		-		-		2.04 (0.97–4.28)	
Yes	-		-		-		1.0	
**Food insufficiency up to 15 years old**		0.13						
Yes	2.15 (0.78–5.9)		-		-		-	
No	1.0		-		-		-	
**Self-rated health**		0.46		**0.008**		0.13		
Bad	1.18 (0.19–7.27)		10.54 (2.01–55.12)		5.1 (0.49–52.85)		-	
Regular	1.96 (0.62–6.14)		0.94 (0.24–3.58)		2.74 (0.86–8.77)		-	
Good	1.0		1.0		1.0		-	
**Subjective well-being**						0.22		
Worse	-		-		2.57 (0.5–13.14)		-	
Better	-		-		1.0		-	
**Global satisfaction with life**				0.08				0.06
Lower	-		4.1 (0.83–20.21)		-		2.26 (0.92–5.57)	
Higher	-		1.0		-		1.0	
**Depressive symptoms**				0.34				
Yes	-		0.51 (0.12–2.16)		-		-	
No	-		1.0		-		-	
**Receiving social support**						0.11		
No	-		-		0.27 (0.05–1.4)		-	
Yes	-		-		1.0		-	
**Offering social support**				0.09				
No	-		6.33 (0.69–57.68)		-		-	
Yes	-		1.0		-		-	
**Home arrangement**				0.38				
Living alone	-		0.12 (0.01–2.6)		-		-	
Spouse only	-		0.76 (0.04–16.23)		-		-	
Offspring	-		0.24 (0.04–1.64)		-		-	
Offspring and grandchildren	-		0.23 (0.03–1.66)		-		-	
Other arrangements	-		1.0		-		-	
**Conviviality most of the time**				0.09				
Living alone	-		0.09 (0.01–0.87)		-		-	
Children and/or adolescents	-		1.36 (0.18–10.01)		-		-	
Seniors	-		1.11 (0.21–5.93)		-		-	
Adults	-		1.0		-		-	

*SABE* Health, well-being and ageing, *ORadj* Adjusted odds ratio, 95 per cent *CI* 95 per cent confidence interval, *ADLs* Activities of daily living, *IADLs* Instrumental activities of daily living

## Discussion

Our findings show that regional differences in Brazil may influence the disability of the older populations. In north-eastern Brazil, the oldest-old people have a larger support network and live longer with their peers, which may contribute to reducing the feeling of loneliness, the prevalence of depression and worse self-perception of health. In the Southeast, financial constraints, subjective poverty, and unmet needs may favour the development of functional limitations between long-lived people.

The high prevalence of IADLs disability between the two studied populations shows that the oldest-old have difficulty in social participation. Also, the higher prevalence of disability for IADLs among oldest-old people in Brejo dos Santos can be explained by the fact that people aged 80 and older living in rural north-eastern Brazil are mostly illiterate, which could make it more difficult to use the telephone, or to use of means of transport, banking and purchasing.

Illiteracy is a historical problem in Brazil. The public schools, until the first half of the twentieth century, were restricted to the privileged classes and the process of expansion of public education did not occur uniformly. Schools were concentrated especially in urban spaces and central regions of cities, while rural areas assumed secondary importance in this process, making illiteracy an essentially rural problem in Brazil. Similarly, the less developed areas of the national territory, such as the Northeast, would comprise most of the country's illiterate population. Illiterate seniors are victims of the educational exclusion experienced in the countryside and in small north-eastern cities and towns that have a life dynamic much closer to the countryside [26].

The literature has already pointed out that education seems to have a very important effect on the chances of suffering functional limitations [27]. Better educated older people tend to have healthier behaviours, better life-long jobs and access to better health information [28]. Unfavourable socioeconomic conditions during childhood can be detrimental to health in adulthood and this disadvantage may persist until ageing [9].

Concerning income, the results of this study indicate that the oldest-old people of São Paulo with the lowest income were more disabled in ADLs and IADLs in both groups, in agreement with previous studies [9, 12]. Also, lower financial status proved to be an independently associated factor with functional impairment of IADLs in the longevous population of São Paulo. However, not considering income sufficient for expenses was an associated factor of functional decline in the SABE-SP sample. However, this finding is noteworthy since in Brejo dos Santos there is a 25.1 percent higher prevalence of older people who reported income of up to one minimum wage.

In the literature, it was found that seniors without financial difficulties were 3.1 times less likely to have severe functional limitations [28]. Due to the greater purchasing power and access to health services, there is a postponement of morbidity among the senior population. Subjective poverty, on the other hand, reflects financial constraints that affect mental health. The existence of potential unmet needs can also lead to functional decline [28]. On the other hand, the income prospects of older people in Brazil are uncertain. The social security system is already suffering the financial pressures of an ageing population. Any changes that reduce the value of benefits will decrease available financial resources [5].

Despite the evident socioeconomic differences, the prevalence of disability for ADLs in the populations of São Paulo and Brejo dos Santos showed a small percentage difference between them, with a higher 4.1 percent among the north-eastern oldest old. A positive association for ADL disability was observed only in the São Paulo population where there was a higher proportion of women in relation to Brejo dos Santos.

There are gender differences in limitations for ADLs, as some diseases are disabling for women but lethal for men. In São Paulo, it was found that disability becomes increasingly significant for women with advancing age, but less so for men as those survive to greater ages tend to have been healthier earlier in life. Measures of disability produced more significant effects for women than men, especially after 80 years of age [29]. Disability was considered a risk factor for male mortality [30]; however, although men have a shorter life expectancy, those who live longer are healthier than the oldest women [31].

Female gender was an associated factor with ADLs in both groups of oldest-old through bivariate analysis, which corroborates previous studies with rural [32] and urban long-lived people [8]. However, among the rural north-eastern long-lived, there was a negative and independent association between females and limitations for ADLs, highlighting differences between this community and others studied in the literature [32].

In Brejo dos Santos, the oldest-old who did not have a partner had more chances of developing disability for ADLs and IADLs. These data are in line with a study conducted with Brazilian long-lived seniors in which those without partners were more likely to have a disability for ADLs and IADLs [33]. However, this association was not observed in the São Paulo sample, possibly due to the population profile which had a lower prevalence of long-lived persons being with partners. These findings agree with other studies in the literature, which showed that spouses and adult children are important sources of social support for older people living in rural areas [34], who are more dependent on family support than those living in urban centres. Social support plays an important role in the health of the seniors and should be strengthened in urban regions [35].

The results of this study show an inverse association between living only with a spouse and functional decline for IADLs in the longevous group living in rural areas, which corroborates the literature [36]. Living alone was negatively associated with disability in ADLs and IADLs, corroborating the findings of a study of people in Sweden aged 80 and older [37]. Those who live alone are more encouraged to deal with daily activities on their own than those who live with their spouses [37]. Some authors have suggested that there may be a selection effect concerning the profile of the seniors who can live alone independently. This association is more present among richer seniors, suggesting that, depending on access to resources, instrumental and caregiver support, and home physical environment, living alone may not be risky [36].

Adverse health outcomes among seniors living alone can be confused by a poor social network that can be considered a “risk factor for social fragility”. Older people who live alone and have poor social networks can be considered socially isolated and this can result in depressive symptoms and anxiety [38]. Social isolation was a predictor of functional decline in a longitudinal study of urban Japanese seniors [15]. Greater ADLs disability was also associated with social isolation among older individuals in India [14].

Loneliness is a significant public health concern among older people, given its association with a wide range of adverse health outcomes [39] and may be associated with functional decline [9]. In the present study, there was a positive association between loneliness and ADL disability only in the group of urban older people. This can be explained by the fact that most of the long-lived in the Brejo dos Santos community live with partners or family members. In a prospective study involving urban Indian seniors, those with feelings of loneliness developed 2.3 times more functional decline for ADLs compared to those who did not report feeling alone [8]. In a longitudinal study conducted in the United States, loneliness was a predictor of functional decline and mortality among participants older than 60 years [13].

Only among the oldest-old in São Paulo, self-rated health was associated with functional impairment, being an independently associated factor of ADL disability [8]. Urban long-lived people who rated their health as bad had 10.54 times more functional deficits for ADLs compared to those who considered their health as good or regular. In older women over 80 years, among the most predictive factors of better functionality for ADLs and IADLs was a good perception of health. The worst self-rated health and depressive symptoms were associated with limitations for ADLs [8], while not having depression was associated with better performance in IADLs [10].

Depressive symptoms were associated with functional decline for ADLs and IADLs in the SABE/SP sample [8, 9]. In line with this result, a prospective study of older adults who were evaluated for approximately 8 years found that depressive symptoms are associated with the development of disabilities in ADLS and IADLs [40]. A six-year longitudinal study involving German seniors aged 75 and older suggested that increased depressive symptoms were associated with subsequent increased disability, and the results showed no association between increased disability and subsequent depressive symptoms. This suggests that early detection of symptoms of depression is important to prevent functional dependence in old age [41].

Resilience can be considered the ability to cope with adversity and, in a qualitative study involving long-lived women, it has been suggested that resilience relates to a strong sense of subjective well-being and life satisfaction [42]. A longitudinal study indicated that resilience protects against the limitations of ADLs and IADLs at an older age, as well as modifying the relationship between the onset of a new chronic condition and subsequent disability. This is important because advancing knowledge of how people face adversity and hardship in adulthood can help clarify how resilience influences the way people adapt to changes associated with ageing and functional limitations [43].

In the present study, the worst subjective well-being was associated with disability in ADLs and IADLs in the São Paulo sample, but this was not repeated for the rural community. Those with long-term with lower subjective well-being developed 5.12 times more disability in ADLs and 2.79 times more disability in IADLs. This data agrees with a study conducted with Israeli seniors aged 75 years and older, where the analyses showed functional decline associated with poorer wellbeing. The authors suggest that differences in well-being levels became more evident when life circumstances changed, including marital status, institutionalisation, and declining health [11].

Urban oldest-old people with lower life satisfaction developed 2.54 times more disability in ADLs. Corroborating this data, the results of a study involving Swedish seniors aged 78 years and older showed that increased disability leads to lower life satisfaction [44]. On the other hand, in this study, there was no association between worse life satisfaction and functional decline among Brejo dos Santos’s oldest old, which distinguishes this rural community from another study in Poland where a lack of satisfaction with life had a significant impact on limitations for ADLs [12].

Life satisfaction may vary among oldest-old aged 80 and over and this may depend on social mores, traditions, and culture. In addition to being under the influence of other dimensions such as sociodemographic characteristics, health and financial status, but also subjective dimensions such as life-related attitudes such as the sense of identity, the purpose of life and resilience. Among Korean elders, the emotional support of family and/or friends attenuated the negative influence of life satisfaction events. Results revealed that life satisfaction trajectories increased with maturity, good family relationships, and increased health perception [45].

Since Brazil is a country of continental dimensions in which there is an accelerated ageing process of its population, longevity is one of the indicators that most have contributed to determining the Municipal Human Development Index (IDHM). In Brejo dos Santos, for example, the IDHM was estimated by 0.619 and, in São Paulo, the value was higher, reaching 0.805 [46]. Older age was associated with disability in both samples, as evidenced in other studies [9, 12, 37]. Over the years, the oldest-old have more difficulties to perform the ADLs as well as the IADLs and increasingly depend on their support network. Understanding trends in disability in the oldest-old is important for planning and policy-making purposes in countries around the world with ageing populations [27]. Public policies and behaviour change interventions should focus on modifiable factors like lifestyle, since some factors such as age, education and income become more difficult to be changed at the end of life.

The association between disability and chronic diseases has been described in recent studies [8, 47]. In a previous study with Brazilian longevous populations, it was shown that inadequate weight, stroke, and joint disease were independently associated with limitations for ADLs, while chronic diseases were associated with difficulties for IADLs in a rural community where there is little variability in socioeconomic conditions [48]. Multimorbidity should be considered as a possible confounding variable in the analyses, since they tend to be associated both with the outcomes and explanatory variables, being a limitation of the present study.

The use of a cross-sectional design allows identifying factors that might be associated with outcome variables but it is not possible to infer causality, which is another limitation of this study. In addition, the evaluation of some independent variables was performed subjectively, being susceptible to memory bias. The results are also potentially affected by an omitted variable bias (OVB). The comparison of the two groups is potentially affected by many variables not included in the analysis, some of them not observable at all. However, these limitations do not compromise the results of this research. Also, the SABE questionnaire uses internationally validated data collection instruments, which are widely used in the scientific literature and allow a broad evaluation of the aspects investigated in this study. To better understand the causal relationship between variables, future studies with a longitudinal or control-case design are recommended.

## Conclusion

Our findings indicate that regional differences in Brazil may influence the disability of older people aged 80 and older. In northeast Brazil, having no partner may contribute to disability for ADLs and IADLs; while, in the longevous population of São Paulo, having a worse self-rated health may contribute to disability for IADLs.

## Supplementary Information

**Additional file 1: Supplementary Table 1.** Comparison of the disability of the oldest-old from Brejo dos Santos/PB and São Paulo/SP in relation to the number of Basic Activities of Daily Living (ADLs) and Instrumental Activities of Daily Living (IADLs) with support.

## Data Availability

The datasets used and/or analysed during the current study are available from the corresponding author on reasonable request.

## Abbreviations

ADLs: Activities of Daily Living
IADLs: Instrumental Activities of Daily Living
SABE: Health, Well-being and Ageing Study (*Saúde**, **Bem-Estar** e **Envelhecimento*)
SABE: PB—Health, Well-being and Ageing Study in Paraíba
SABE: SP—Health, Well-being and Ageing Study in São Paulo
