# Supplementary material for: Psychosocial aspects and support networks associated with disability in two longevous populations in Brazil: a cross-sectional study
Source: BMC Geriatr. 2022 Feb 9;22:110. doi: 10.1186/s12877-022-02810-4 (PMC8826700; doi:10.1186/s12877-022-02810-4)
Supplement: Supplementary file 1 — Additional file 1: Supplementary Table 1. Comparison of the disability of the oldest-old from Brejo dos Santos/PB and São Paulo/SP in relation to the number of Basic Activities of Daily Living (ADLs) and Instrumental Activities of Daily Living (IADLs) with support. [file 12877_2022_2810_MOESM1_ESM.docx]

| **Supplementary Table 1.** Comparison of the disability of the oldest-old from Brejo dos Santos/PB and São Paulo/SP in relation to the number of Basic Activities of Daily Living (ADLs) and Instrumental Activities of Daily Living (IADLs) with support. | | | | | | | | |
| --- | --- | --- | --- | --- | --- | --- | --- | --- |
|  | | | | | | | | |
| **Number of activities with support** | **ADLs** | | | | **IADLs** | | | |
|  | **SABE - PB** | | **SABE-SP** | | **SABE - PB** | | **SABE-SP** | |
|  | **n** | **%** | **n** | **%** | **n** | **%** | **n** | **%** |
| 0 | 122 | 69.30 | 171 | 79.90 | 23 | 15.00 | 63 | 29.20 |
| 1 | 12 | 6.80 | 9 | 4.20 | 25 | 16.30 | 24 | 11.10 |
| 2 | 7 | 4.00 | 10 | 4.70 | 27 | 17.60 | 28 | 13.00 |
| 3 | 5 | 2.80 | 3 | 1.40 | 13 | 8.50 | 10 | 4.60 |
| 4 | 6 | 3.40 | 4 | 1.90 | 16 | 10.50 | 19 | 8.80 |
| 5 | 5 | 2.80 | 4 | 1.90 | 11 | 7.20 | 14 | 6.50 |
| 6 | 10 | 5.70 | 6 | 2.80 | 20 | 13.10 | 21 | 9.70 |
| 7 | 9 | 5.10 | 7 | 3.30 | 18 | 11.80 | 37 | 17.10 |
| SABE Study, Brejo dos Santos/PB, Brazil, 2017 and São Paulo/SP, Brazil, 2015. | | | | | | | | |
